# 3-(3-Fluoro­phenyl­sulfin­yl)-2,5,7-trimethyl-1-benzofuran

**DOI:** 10.1107/S1600536811018654

**Published:** 2011-05-20

**Authors:** Hong Dae Choi, Pil Ja Seo, Byeng Wha Son, Uk Lee

**Affiliations:** a Department of Chemistry, Dongeui University, San 24 Kaya-dong Busanjin-gu, Busan 614-714, Republic of Korea; b Department of Chemistry, Pukyong National University, 599-1 Daeyeon 3-dong, Nam-gu, Busan 608-737, Republic of Korea

## Abstract

In the title compound, C_17_H_15_FO_2_S, the 3-fluoro­phenyl ring makes a dihedral angle of 86.89 (4)° with the mean plane of the benzofuran fragment. In the crystal, mol­ecules are linked by weak inter­molecular C—H⋯O hydrogen bonds. The crystal structure also exhibits a slipped π–π inter­action between the furan rings of neighbouring mol­ecules [centroid–centroid distance = 3.719 (2) Å, inter­planar distance = 3.475 (2) Å and slippage = 1.325 Å].

## Related literature

For the biological activity of benzofuran compounds, see: Aslam *et al.* (2009 ▶); Galal *et al.* (2009 ▶); Khan *et al.* (2005 ▶). For natural products with benzofuran rings, see: Akgul & Anil (2003 ▶); Soekamto *et al.* (2003 ▶). For structural studies of related 3-(4-fluoro­phenyl­sulfin­yl)-2,5-dimethyl-1-benzofuran derivatives, see: Choi *et al.* (2010*a* ▶,*b* ▶).
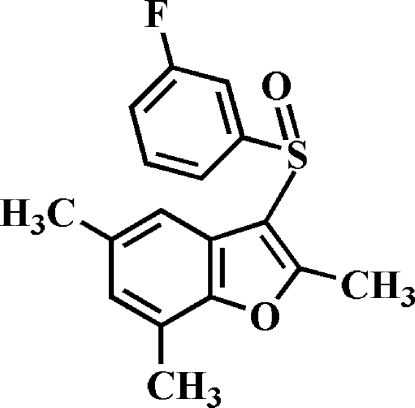


## Experimental

### 

#### Crystal data

C_17_H_15_FO_2_S
*M* *_r_* = 302.35
Triclinic, 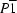

*a* = 6.2942 (2) Å
*b* = 11.1162 (4) Å
*c* = 11.8021 (6) Å
α = 110.701 (3)°
β = 100.176 (3)°
γ = 103.621 (2)°
*V* = 719.39 (5) Å^3^
*Z* = 2
Mo *K*α radiation
μ = 0.24 mm^−1^
*T* = 296 K
0.29 × 0.25 × 0.21 mm

#### Data collection

Bruker SMART APEXII CCD diffractometer
Absorption correction: multi-scan (*SADABS*; Bruker, 2009 ▶) *T*_min_ = 0.935, *T*_max_ = 0.951
12696 measured reflections
3301 independent reflections
2760 reflections with *I* > 2σ(*I*)
*R*_int_ = 0.122

#### Refinement

*R*[*F* ^2^ > 2σ(*F* ^2^)] = 0.047
*wR*(*F* ^2^) = 0.133
*S* = 1.07
3301 reflections
193 parameters
H-atom parameters constrained
Δρ_max_ = 0.52 e Å^−3^
Δρ_min_ = −0.44 e Å^−3^

### 

Data collection: *APEX2* (Bruker, 2009 ▶); cell refinement: *SAINT* (Bruker, 2009 ▶); data reduction: *SAINT*; program(s) used to solve structure: *SHELXS97* (Sheldrick, 2008 ▶); program(s) used to refine structure: *SHELXL97* (Sheldrick, 2008 ▶); molecular graphics: *ORTEP-3* (Farrugia, 1997 ▶) and *DIAMOND* (Brandenburg, 1998 ▶); software used to prepare material for publication: *SHELXL97*.

## Supplementary Material

Crystal structure: contains datablocks global, I. DOI: 10.1107/S1600536811018654/ez2242sup1.cif

Structure factors: contains datablocks I. DOI: 10.1107/S1600536811018654/ez2242Isup2.hkl

Supplementary material file. DOI: 10.1107/S1600536811018654/ez2242Isup3.cml

Additional supplementary materials:  crystallographic information; 3D view; checkCIF report

## Figures and Tables

**Table 1:** Hydrogen-bond geometry (Å, °)

*D*—H⋯*A*	*D*—H	H⋯*A*	*D*⋯*A*	*D*—H⋯*A*
C13—H13⋯O2^i^	0.93	2.51	3.227 (2)	134

Symmetry code: (i) 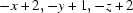.

**Fig. 1.**
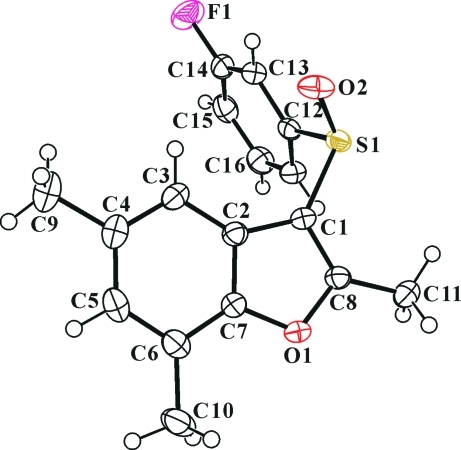
The molecular structure of the title compound with the atom numbering scheme. Displacement ellipsoids are drawn at the 50% probability level. H atoms are presented as a small spheres of arbitrary radius.

**Fig. 2.**
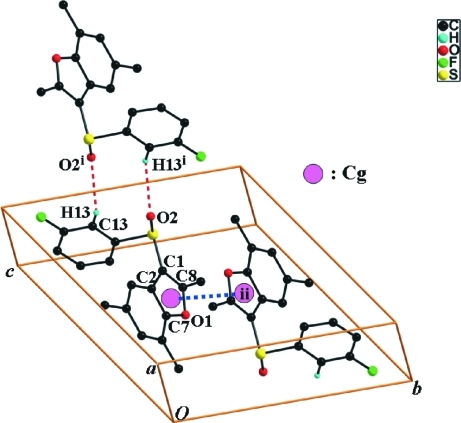
A view of the C—H···O and π–π interactions (dotted lines) in the crystal structure of the title compound. [Symmetry codes: (i) - x + 2, - y + 1, - z + 2 ; (ii) - x + , - y + 1, - z + 1.]

## supplementary crystallographic information

### Comment

Many compounds containing a benzofuran ring system exhibit interesting pharmacological properties such as antibacterial and antifungal, antitumor and antiviral, and antimicrobial activities (Aslam *et al.*, 2009, Galal *et al.*, 2009, Khan *et al.*, 2005). These compounds occur in a wide range of natural products (Akgul & Anil, 2003; Soekamto *et al.*, 2003). As a part of our study of the substituent effect on the solid state structures of 3-(4-fluorophenylsulfinyl)-2,5-dimethyl-1-benzofuran analogues (Choi *et al.*, 2010*a*,*b*), we report the crystal structure of the title compound.

In the title compound (Fig. 1), the benzofuran unit is essentially planar, with a mean deviation of 0.007 (1) Å from the least-squares plane defined by the nine constituent atoms. The 3-fluorophenyl ring makes a dihedral angle of 86.89 (4)° with the mean plane of the benzofuran fragment. The crystal packing (Fig. 2) is stabilized by weak intermolecular C—H···O hydrogen bonds between a 3-fluorophenyl H atom and the O atom of the sulfinyl group (Table 1; C13—H13···O2^i^). The crystal packing (Fig. 2) is further stabilized by a weak slipped π–π interaction between the furan rings of neighbouring molecules, with a Cg···Cg^ii^ distance of 3.719 (2) Å and an interplanar distance of 3.475 (2) Å resulting in a slippage of 1.325 Å (Cg is the centroid of the C1/C2/C7/O1/C8 furan ring).

### Experimental

77% 3-chloroperoxybenzoic acid (291 mg, 1.3 mmol) was added in small portions to a stirred solution of 3-(3-fluorophenylsulfanyl)-2,5,7-trimethyl-1-benzofuran (342 mg, 1.2 mmol) in dichloromethane (40 mL) at 273 K. After being stirred at room temperature for 4h, the mixture was washed with saturated sodium bicarbonate solution and the organic layer was separated, dried over magnesium sulfate, filtered and concentrated at reduced pressure. The residue was purified by column chromatography (hexane–ethyl acetate, 1:1 v/v) to afford the title compound as a colorless solid [yield 75%, m.p. 434–435 K; *R*_f_ = 0.72 (hexane–ethyl acetate, 1:1 v/v)]. Single crystals suitable for X-ray diffraction were prepared by slow evaporation of a solution of the title compound in ethyl acetate at room temperature.

### Refinement

All H atoms were positioned geometrically and refined using a riding model, with C—H = 0.95 Å for aryl and 0.96 Å for methyl H atoms. *U*_iso_(H) =1.2*U*_eq_(C) for aryl and 1.5*U*_eq_(C) for methyl H atoms.

### Crystal data

**Table:**

C_17_H_15_FO_2_S	*Z* = 2
*M**_r_* = 302.35	*F*(000) = 316
Triclinic, *P*1	*D*_x_ = 1.396 Mg m^−^^3^
Hall symbol: -P 1	Mo *K*α radiation, λ = 0.71073 Å
*a* = 6.2942 (2) Å	Cell parameters from 5769 reflections
*b* = 11.1162 (4) Å	θ = 2.2–27.5°
*c* = 11.8021 (6) Å	µ = 0.24 mm^−^^1^
α = 110.701 (3)°	*T* = 296 K
β = 100.176 (3)°	Block, colourless
γ = 103.621 (2)°	0.29 × 0.25 × 0.21 mm
*V* = 719.39 (5) Å^3^	

### Data collection

**Table:**

Bruker SMART APEXII CCD diffractometer	3301 independent reflections
Radiation source: rotating anode	2760 reflections with *I* > 2σ(*I*)
graphite multilayer	*R*_int_ = 0.122
Detector resolution: 10.0 pixels mm^-1^	θ_max_ = 27.5°, θ_min_ = 1.9°
φ and ω scans	*h* = −8→8
Absorption correction: multi-scan (*SADABS*; Bruker, 2009)	*k* = −14→14
*T*_min_ = 0.935, *T*_max_ = 0.951	*l* = −15→15
12696 measured reflections	

### Refinement

**Table:**

Refinement on *F*^2^	Primary atom site location: structure-invariant direct methods
Least-squares matrix: full	Secondary atom site location: difference Fourier map
*R*[*F*^2^ > 2σ(*F*^2^)] = 0.047	Hydrogen site location: difference Fourier map
*wR*(*F*^2^) = 0.133	H-atom parameters constrained
*S* = 1.07	*w* = 1/[σ^2^(*F*_o_^2^) + (0.0679*P*)^2^ + 0.086*P*] where *P* = (*F*_o_^2^ + 2*F*_c_^2^)/3
3301 reflections	(Δ/σ)_max_ < 0.001
193 parameters	Δρ_max_ = 0.52 e Å^−^^3^
0 restraints	Δρ_min_ = −0.44 e Å^−^^3^

### Special details

**Table:**

Geometry. All esds (except the esd in the dihedral angle between two l.s. planes) are estimated using the full covariance matrix. The cell esds are taken into account individually in the estimation of esds in distances, angles and torsion angles; correlations between esds in cell parameters are only used when they are defined by crystal symmetry. An approximate (isotropic) treatment of cell esds is used for estimating esds involving l.s. planes.
Refinement. Refinement of F^2^ against ALL reflections. The weighted R-factor wR and goodness of fit S are based on F^2^, conventional R-factors R are based on F, with F set to zero for negative F^2^. The threshold expression of F^2^ > 2sigma(F^2^) is used only for calculating R-factors(gt) etc. and is not relevant to the choice of reflections for refinement. R-factors based on F^2^ are statistically about twice as large as those based on F, and R- factors based on ALL data will be even larger.

### Fractional atomic coordinates and isotropic or equivalent isotropic displacement parameters (Å^2^)

**Table:**

	*x*	*y*	*z*	*U*_iso_*/*U*_eq_	
S1	0.61779 (7)	0.50213 (4)	0.83763 (4)	0.02605 (15)	
O1	0.2342 (2)	0.38830 (12)	0.49387 (10)	0.0275 (3)	
O2	0.8652 (2)	0.52089 (14)	0.85568 (12)	0.0361 (3)	
F1	0.7099 (2)	0.14993 (13)	1.00630 (12)	0.0517 (4)	
C1	0.4798 (3)	0.42715 (17)	0.67501 (15)	0.0249 (3)	
C2	0.5132 (3)	0.31895 (16)	0.57616 (15)	0.0250 (3)	
C3	0.6547 (3)	0.23900 (18)	0.56868 (17)	0.0303 (4)	
H3	0.7618	0.2516	0.6406	0.036*	
C4	0.6325 (3)	0.14076 (18)	0.45232 (18)	0.0342 (4)	
C5	0.4696 (3)	0.12330 (18)	0.34451 (18)	0.0350 (4)	
H5	0.4573	0.0561	0.2670	0.042*	
C6	0.3262 (3)	0.20148 (18)	0.34811 (16)	0.0307 (4)	
C7	0.3566 (3)	0.29841 (16)	0.46668 (15)	0.0256 (4)	
C8	0.3133 (3)	0.46537 (17)	0.62103 (15)	0.0253 (3)	
C9	0.7843 (4)	0.0524 (2)	0.4405 (2)	0.0470 (5)	
H9A	0.9238	0.0970	0.4290	0.070*	
H9B	0.7075	−0.0332	0.3691	0.070*	
H9C	0.8177	0.0373	0.5160	0.070*	
C10	0.1525 (4)	0.1847 (2)	0.23408 (17)	0.0417 (5)	
H10A	0.0034	0.1632	0.2459	0.063*	
H10B	0.1568	0.1125	0.1608	0.063*	
H10C	0.1863	0.2678	0.2226	0.063*	
C11	0.2019 (3)	0.56740 (18)	0.67277 (17)	0.0324 (4)	
H11A	0.0490	0.5220	0.6687	0.049*	
H11B	0.1982	0.6205	0.6241	0.049*	
H11C	0.2863	0.6259	0.7591	0.049*	
C12	0.5032 (3)	0.35709 (16)	0.86983 (14)	0.0243 (3)	
C13	0.6565 (3)	0.30906 (17)	0.92600 (15)	0.0280 (4)	
H13	0.8132	0.3478	0.9441	0.034*	
C14	0.5646 (3)	0.20107 (18)	0.95355 (17)	0.0326 (4)	
C15	0.3363 (3)	0.14339 (18)	0.93105 (17)	0.0352 (4)	
H15	0.2814	0.0706	0.9513	0.042*	
C16	0.1877 (3)	0.19579 (19)	0.87727 (18)	0.0352 (4)	
H16	0.0313	0.1583	0.8618	0.042*	
C17	0.2699 (3)	0.30294 (18)	0.84667 (17)	0.0309 (4)	
H17	0.1703	0.3384	0.8110	0.037*	

### Atomic displacement parameters (Å^2^)

**Table:**

	*U*^11^	*U*^22^	*U*^33^	*U*^12^	*U*^13^	*U*^23^
S1	0.0285 (3)	0.0248 (2)	0.0201 (2)	0.00624 (17)	0.00117 (16)	0.00820 (17)
O1	0.0280 (6)	0.0311 (6)	0.0233 (6)	0.0126 (5)	0.0020 (5)	0.0117 (5)
O2	0.0248 (6)	0.0437 (7)	0.0335 (7)	0.0014 (5)	−0.0012 (5)	0.0196 (6)
F1	0.0560 (8)	0.0466 (7)	0.0606 (8)	0.0225 (6)	0.0038 (6)	0.0334 (7)
C1	0.0251 (8)	0.0269 (8)	0.0228 (8)	0.0100 (6)	0.0033 (6)	0.0111 (7)
C2	0.0256 (8)	0.0249 (8)	0.0247 (8)	0.0080 (7)	0.0053 (6)	0.0115 (7)
C3	0.0283 (9)	0.0320 (9)	0.0349 (9)	0.0141 (7)	0.0078 (7)	0.0163 (8)
C4	0.0365 (10)	0.0290 (9)	0.0430 (11)	0.0148 (8)	0.0173 (8)	0.0159 (8)
C5	0.0454 (11)	0.0257 (8)	0.0300 (9)	0.0093 (8)	0.0147 (8)	0.0067 (8)
C6	0.0352 (9)	0.0274 (8)	0.0255 (8)	0.0058 (7)	0.0078 (7)	0.0098 (7)
C7	0.0267 (8)	0.0248 (8)	0.0250 (8)	0.0091 (7)	0.0059 (7)	0.0104 (7)
C8	0.0267 (8)	0.0264 (8)	0.0222 (8)	0.0084 (7)	0.0040 (6)	0.0110 (7)
C9	0.0534 (13)	0.0413 (11)	0.0591 (13)	0.0285 (10)	0.0272 (11)	0.0216 (11)
C10	0.0533 (12)	0.0370 (10)	0.0235 (9)	0.0065 (9)	0.0003 (8)	0.0101 (8)
C11	0.0341 (10)	0.0335 (9)	0.0336 (9)	0.0173 (8)	0.0086 (8)	0.0148 (8)
C12	0.0290 (8)	0.0238 (8)	0.0164 (7)	0.0082 (6)	0.0034 (6)	0.0058 (6)
C13	0.0275 (8)	0.0291 (8)	0.0241 (8)	0.0100 (7)	0.0031 (7)	0.0088 (7)
C14	0.0439 (10)	0.0287 (8)	0.0261 (8)	0.0176 (8)	0.0051 (8)	0.0109 (7)
C15	0.0465 (11)	0.0263 (9)	0.0292 (9)	0.0071 (8)	0.0104 (8)	0.0104 (7)
C16	0.0306 (9)	0.0335 (9)	0.0360 (10)	0.0046 (8)	0.0098 (8)	0.0116 (8)
C17	0.0270 (8)	0.0339 (9)	0.0292 (9)	0.0107 (7)	0.0038 (7)	0.0116 (8)

### Geometric parameters (Å, °)

**Table:**

S1—O2	1.4876 (13)	C9—H9A	0.9600
S1—C1	1.7527 (16)	C9—H9B	0.9600
S1—C12	1.8017 (17)	C9—H9C	0.9600
O1—C8	1.3639 (19)	C10—H10A	0.9600
O1—C7	1.3851 (19)	C10—H10B	0.9600
F1—C14	1.357 (2)	C10—H10C	0.9600
C1—C8	1.360 (2)	C11—H11A	0.9600
C1—C2	1.437 (2)	C11—H11B	0.9600
C2—C7	1.389 (2)	C11—H11C	0.9600
C2—C3	1.391 (2)	C12—C17	1.386 (2)
C3—C4	1.378 (3)	C12—C13	1.386 (2)
C3—H3	0.9300	C13—C14	1.377 (3)
C4—C5	1.407 (3)	C13—H13	0.9300
C4—C9	1.513 (2)	C14—C15	1.363 (3)
C5—C6	1.389 (3)	C15—C16	1.388 (3)
C5—H5	0.9300	C15—H15	0.9300
C6—C7	1.378 (2)	C16—C17	1.377 (3)
C6—C10	1.499 (2)	C16—H16	0.9300
C8—C11	1.479 (2)	C17—H17	0.9300
			
O2—S1—C1	108.38 (8)	H9A—C9—H9C	109.5
O2—S1—C12	105.93 (7)	H9B—C9—H9C	109.5
C1—S1—C12	97.25 (7)	C6—C10—H10A	109.5
C8—O1—C7	106.64 (12)	C6—C10—H10B	109.5
C8—C1—C2	107.69 (14)	H10A—C10—H10B	109.5
C8—C1—S1	123.27 (13)	C6—C10—H10C	109.5
C2—C1—S1	129.04 (12)	H10A—C10—H10C	109.5
C7—C2—C3	119.25 (15)	H10B—C10—H10C	109.5
C7—C2—C1	104.76 (13)	C8—C11—H11A	109.5
C3—C2—C1	135.99 (15)	C8—C11—H11B	109.5
C4—C3—C2	118.52 (16)	H11A—C11—H11B	109.5
C4—C3—H3	120.7	C8—C11—H11C	109.5
C2—C3—H3	120.7	H11A—C11—H11C	109.5
C3—C4—C5	119.93 (16)	H11B—C11—H11C	109.5
C3—C4—C9	119.97 (17)	C17—C12—C13	121.95 (16)
C5—C4—C9	120.09 (18)	C17—C12—S1	120.18 (12)
C6—C5—C4	123.21 (17)	C13—C12—S1	117.72 (13)
C6—C5—H5	118.4	C14—C13—C12	116.56 (16)
C4—C5—H5	118.4	C14—C13—H13	121.7
C7—C6—C5	114.34 (16)	C12—C13—H13	121.7
C7—C6—C10	121.75 (17)	F1—C14—C15	118.44 (17)
C5—C6—C10	123.91 (17)	F1—C14—C13	118.02 (17)
C6—C7—O1	124.90 (15)	C15—C14—C13	123.54 (16)
C6—C7—C2	124.74 (15)	C14—C15—C16	118.45 (17)
O1—C7—C2	110.36 (14)	C14—C15—H15	120.8
C1—C8—O1	110.54 (14)	C16—C15—H15	120.8
C1—C8—C11	133.15 (16)	C17—C16—C15	120.52 (17)
O1—C8—C11	116.28 (13)	C17—C16—H16	119.7
C4—C9—H9A	109.5	C15—C16—H16	119.7
C4—C9—H9B	109.5	C16—C17—C12	118.94 (16)
H9A—C9—H9B	109.5	C16—C17—H17	120.5
C4—C9—H9C	109.5	C12—C17—H17	120.5
			
O2—S1—C1—C8	−138.58 (15)	C1—C2—C7—C6	−178.81 (16)
C12—S1—C1—C8	111.91 (16)	C3—C2—C7—O1	−179.52 (15)
O2—S1—C1—C2	41.89 (18)	C1—C2—C7—O1	0.63 (19)
C12—S1—C1—C2	−67.61 (17)	C2—C1—C8—O1	0.7 (2)
C8—C1—C2—C7	−0.79 (19)	S1—C1—C8—O1	−178.92 (12)
S1—C1—C2—C7	178.79 (14)	C2—C1—C8—C11	178.47 (19)
C8—C1—C2—C3	179.4 (2)	S1—C1—C8—C11	−1.1 (3)
S1—C1—C2—C3	−1.0 (3)	C7—O1—C8—C1	−0.30 (19)
C7—C2—C3—C4	−0.7 (3)	C7—O1—C8—C11	−178.49 (15)
C1—C2—C3—C4	179.12 (19)	O2—S1—C12—C17	−172.33 (13)
C2—C3—C4—C5	0.1 (3)	C1—S1—C12—C17	−60.80 (15)
C2—C3—C4—C9	179.70 (17)	O2—S1—C12—C13	12.06 (14)
C3—C4—C5—C6	0.2 (3)	C1—S1—C12—C13	123.59 (13)
C9—C4—C5—C6	−179.39 (19)	C17—C12—C13—C14	2.3 (2)
C4—C5—C6—C7	0.1 (3)	S1—C12—C13—C14	177.84 (12)
C4—C5—C6—C10	179.67 (18)	C12—C13—C14—F1	178.73 (15)
C5—C6—C7—O1	179.93 (16)	C12—C13—C14—C15	−1.5 (3)
C10—C6—C7—O1	0.3 (3)	F1—C14—C15—C16	179.91 (16)
C5—C6—C7—C2	−0.7 (3)	C13—C14—C15—C16	0.1 (3)
C10—C6—C7—C2	179.67 (17)	C14—C15—C16—C17	0.5 (3)
C8—O1—C7—C6	179.21 (16)	C15—C16—C17—C12	0.3 (3)
C8—O1—C7—C2	−0.23 (19)	C13—C12—C17—C16	−1.8 (3)
C3—C2—C7—C6	1.0 (3)	S1—C12—C17—C16	−177.22 (13)

### Hydrogen-bond geometry (Å, °)

**Table:**

*D*—H···*A*	*D*—H	H···*A*	*D*···*A*	*D*—H···*A*
C13—H13···O2^i^	0.93	2.51	3.227 (2)	134

Symmetry codes: (i) −*x*+2, −*y*+1, −*z*+2.
